# Favorable outcome of Epstein-Barr virus-associated B-cell lymphoproliferative disorder complicated by immunoglobulin G4-related disease treated with rituximab-based therapy: a case report

**DOI:** 10.1186/s13256-016-1009-1

**Published:** 2016-08-24

**Authors:** Koki Ueda, Kazuhiko Ikeda, Kazuei Ogawa, Masumi Sukegawa, Takahiro Sano, Satoshi Kimura, Osamu Suzuki, Yuko Hashimoto, Yasuchika Takeishi

**Affiliations:** 1 Department of Cardiology and Hematology, Fukushima Medical University, 1 Hikarigaoka, Fukushima, 960-1295 Japan; 2 Department of Blood Transfusion and Transplantation Immunology, Fukushima Medical University, 1 Hikarigaoka, Fukushima, 960-1295 Japan; 3 Department of Pathology and Diagnostic Pathology, Fukushima Medical University, 1 Hikarigaoka, Fukushima, 960-1295 Japan

**Keywords:** B cell, Epstein-Barr virus, IgG4-related disease, Rituximab, Case report

## Abstract

**Background:**

After acute infection of Epstein-Barr virus, Epstein-Barr virus-infected B cells survive but usually do not show clonal proliferation. However, Epstein-Barr virus-infected B cells occasionally acquire a proliferative capacity that provokes clonal lymphoproliferative disorders. We herein present a case with Epstein-Barr virus-infected CD30+ B cell and immunoglobulin G4+ plasmacytoid cell proliferation in the lymph nodes, suggesting a pathological and clinical interaction between Epstein-Barr virus-associated B-cell lymphoproliferative disorders and immunoglobulin G4-related disease. Immunoglobulin G4-related disease has been recognized as a benign disease with proliferation of IgG4-related disease+ plasmacytoid cells. Several studies have recently reported the coexistence of immunoglobulin G4-related disease+ plasmacytoid cells with Epstein-Barr virus-infected B cells in lymph nodes in some immunoglobulin G4-related disease cases. However, the pathogenic role of the clonal proliferation of Epstein-Barr virus-infected B cells in immunoglobulin G4-related disease, as well as the treatments for patients with both Epstein-Barr virus-infected B cells and immunoglobulin G4-related disease, have never been discussed.

**Case Presentation:**

A 50-year-old Japanese man was referred to us for persistent fatigue and lymphadenopathy. His blood examination showed elevated IgG4, and detected high levels of Epstein-Barr virus DNA. A lymph node biopsy revealed IgG4+ plasmacytoid cells and infiltration of large lymphoid cells, which were positive for CD20, CD30, Epstein-Barr virus-related late membrane protein 1, and Epstein-Barr virus-encoded RNA, and were negative for IgG4. Based on the diagnosis of both Epstein-Barr virus-associated B-cell lymphoproliferative disorder and IgG4-related disease, the patient received eight cycles of rituximab combined with cyclophosphamide and prednisolone, which resulted in the complete disappearance of lymphadenopathy. Moreover, his serum IgG4 level was significantly reduced, and plasma Epstein-Barr virus DNA became undetectable. Although prednisolone was transiently administered in each cycle of immunochemotherapy, the therapeutic effect has persisted for Epstein-Barr virus-associated B-cell lymphoproliferative disorder and IgG4-related disease as of 1 year after finishing treatment.

**Conclusions:**

In the present case, clinical presentation and pathological findings revealed that Epstein-Barr virus-associated B-cell lymphoproliferative disorder coexisted with IgG4-related disease. Although several studies have described the relationship between Epstein-Barr virus-infected B cells and IgG4-related disease, this is the first report of a patient whose plasma Epstein-Barr virus DNA level, which correlated with the disease statuses of both diseases, was monitored. Moreover, rituximab-based immunochemotherapy was highly effective for both diseases. Our findings are suggestive for establishing a novel treatment strategy for IgG4-related disorders associated with chronic Epstein-Barr virus infection.

## Background

Persistent infection of Epstein-Barr virus (EBV) in B cells occasionally evokes a variety of lymphoproliferative disorders (LPDs). These include malignant lymphomas such as diffuse large B-cell lymphoma (DLBCL) and Hodgkin’s lymphoma. However, polymorphous B-cell LPDs resembling reactive lymphoid hyperplasia or nodal/extranodal polymorphic LPD are also observed in infected individuals [1, 2]. In most cases, after acute infection of EBV, the infected B cells continue to be present in small quantities and do not usually give rise to LPD. However, systemic or localized immunodeficiency may transform EBV-infected B cells into clonal or malignant status [3]. Given that transformation of EBV-infected B cells arises on the background of immunocompromised conditions, it is conceivable that another disease caused by an immunological disorder could coexist in the same lesion as the EBV-associated B-cell LPDs.

Immunoglobulin (Ig) G4-related disease (IgG4-RD) is a newly defined syndrome characterized by sclerosing and mass-forming changes of various lymphoid and non-lymphoid tissues. IgG4-RD are caused by invasion of IgG4-producing plasma cells and/or B cells that lead to fibroinflammatory conditions, and immunological disorder is thought to be a key factor of the pathogenesis of IgG4-RD [4]. To date, only two reports have shown that IgG4-RD may be associated with chronic infection of EBV [2, 3]. Takahashi *et al*. recently described a single case of concurrent IgG4-related lymphadenopathy and EBV infection [5]. Subsequently, it was reported that 18 of 31 (58 %) IgG4-related lymphadenopathy specimens harbored EBV-encoded RNA (EBER)^+^ cells, whereas EBER^+^ cells were detected in only 4 of 22 (18 %) reactive lymphoid hyperplasia specimens [6]. Although these reports shed light on the morphological coexistence of EBV-infected B cells and IgG4-producing cells in the lymph nodes, the interaction between the systemic status of EBV infection and IgG4-RD remains to be elucidated.

In EBV-associated B-cell LPDs, neoplastic EBV-positive cells often co-express CD30 [7, 8]. The combination of CD30 expression and EBV infection in neoplastic B cells results in poor outcome in EBV-associated B-cell LPDs, most evidently in DLBCL [9]. Here, we report a case of successful treatment with rituximab-combined immunochemotherapy in a patient with IgG4-RD complicated with polymorphous EBV-associated B-cell LPD, and discuss the pathogenesis and treatment of the disease.

## Case presentation

**Fig. 1:**
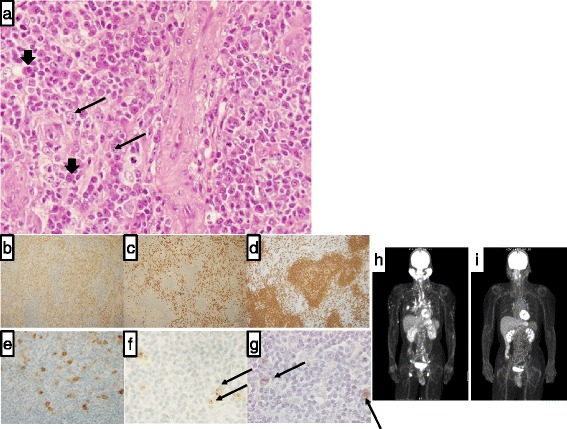
Histological, immunohistochemical and imaging findings of the case. **a** Hematoxylin and eosin staining of the inguinal node (×400). In the **a**, **f**, **g**, plasmacytoid cells are indicated by *large arrows*, and large lymphoid cells are indicated by *small arrows*. **b** Immunoglobulin G, **c** immunoglobulin G4, **d** CD20, **e** CD30, **f** latent membrane protein 1, **g** Epstein-Barr virus-encoded RNA in situ hybridization staining. **h** Fluorodeoxy glucose positron emission tomography-computed tomography at the diagnosis. **i** Fluorodeoxy glucose positron emission tomography-computed tomography after treatment

A 50-year-old Japanese man was referred to our hospital with fatigue and swelling of the parotid glands and inguinal nodes. His blood count was normal, and no major organ dysfunction was detected by biochemical tests. However, markedly elevated values of serum IgG (6188 mg/dL; normal range 870–1700 mg/dL) and IgG4 (2970 mg/dL; normal range 4.8–105 mg/dL) were noted. Soluble-IL2 receptor (sIL2-R) (2130 U/mL; normal range 124–466 U/mL) was also increased. A high level of EBV DNA was detected in the serum plasma (3.0 × 10^4^ copies/mL), with elevated IgG antibodies to EBV viral capsid antigen (VCA) (detectable at a 1:1280 titer) and early antigen (EA) (detectable at a 1:160 titer). Anti-EB nuclear antigen (EBNA) antibody was also detected (detectable at a 1:80 titer), indicating a chronic active EBV infection. Antibodies to hepatitis B virus, hepatitis C virus, human immunodeficiency virus, and human T-cell lymphotrophic virus type I were negative. Systemic positron emission tomography-computed tomography (PET-CT) with fluorodeoxy glucose (FDG) revealed an uptake of FDG in our patient’s enlarged parotid glands and kidneys, as well as the cervical, mediastinal, and inguinal lymph nodes (Fig. 1h). An inguinal lymph node biopsy was performed. Histologically, the lymph node architecture was almost preserved except for an expanded interfollicular zone. Both small- or medium-sized plasmacytoid cells and large lymphoid cells proliferated in the interfollicular zone with formations of small vessels (Fig. 1a). Most plasmacytoid cells were positive for IgG/IgG4 and CD20 (Fig. 1b, c, d), fulfilling the histological criteria for IgG4-related disease [10]. In contrast, large lymphoid cells were positive for CD20 and CD30 (Fig. 1d, e). These large lymphoid cells were also positive for latent membrane protein (LMP)-1 and EBER (Fig. 1f, g), indicating latency III infection of EBV [11]. Since EBV+ LPDs consist of EBV-associated reactive lymphoid hyperplasia, EBV-associated polymorphic lymphoproliferative disease, and EBV+ DLBCL [12], we evaluated the rearrangement of Ig heavy- and light-chains by polymerase chain reaction. This analysis did not show clonal rearrangement, suggesting that the B-cell proliferation of this patient belongs to a subtype such as EBV-associated polymorphic lymphoproliferative disease rather than EBV+ DLBCL. Thus, diagnoses of both IgG4-RD and polymorphous EBV-associated B-cell LPD were made. On the other hand, there was no evidence of EBV infection in the parotid gland specimen, although it was heavily infiltrated with IgG/IgG4-positive plasmacytoid cells, but not large lymphoid cells. Ig-kappa+ cells and Ig-lambda+ cells were similarly distributed, indicating polyclonal proliferation of these plasmacytoid cells. Thus, we worked up his baseline immunodeficiency, which can potentially contribute to the development of IgG4-RD or chronic EBV infection. However, the proportions of CD3+, CD4+, and CD8+ lymphocytes were normal, and a review of the patient’s medical records did not show any evidence of genetic or inherited immunodeficiency in his family.

**Fig. 2:**
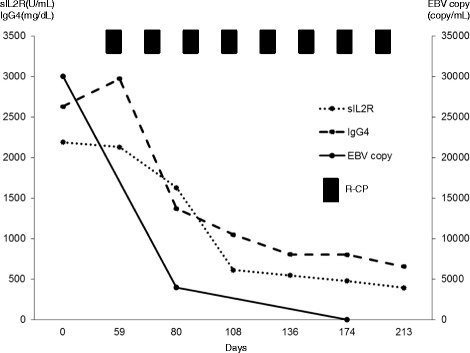
Clinical course of the patient. *R-CP* indicates immunochemotherapy with rituximab, cyclophosphamide and prednisolone; sIL2R, soluble interleukin 2 receptor; IgG4, immunoglobulin G4; EBV, Epstein-Barr virus

Considering the massive proliferation of EBV-infected large B cells with the IgG4^+^ plasmacytoid cells, he was treated with rituximab (375 mg/m^2^), oral cyclophosphamide (100 mg/day for 5 days), and prednisolone (40 mg/day for 5 days) biweekly. After eight courses of therapy, FDG uptake disappeared and the size of the parotid gland, as well as the lymph nodes, was normalized in PET-CT (Fig. 1i). In addition, plasma EBV DNA became undetectable and sIL2-R (395 U/mL) and IgG4 (655 mg/dL) were reduced (Fig. 2). Despite our recommendation, our patient did not receive maintenance therapy, because he was concerned about potential adverse reactions due to the consecutive administration of steroids.

## Discussion

There have been few studies describing the relationship between IgG4-RD and EBV infection [5, 6]. According to these studies, EBV is more frequently positive in lymph nodes than in the extranodal lesion of IgG4-RD. Similarly, in the present case, EBV-infected CD20^+^ lymphoid cells were found only in the inguinal lymph nodes, not in the parotid glands. Therefore, it may be better to evaluate lymph nodes rather than extranodal tissues to detect EBV-infected cells in IgG4-RD, even if IgG4-RD is already evident in extranodal tissues. In addition, our patient did not show any evidence of immunodeficiency. It is well known that immunodeficient conditions that correlate with clonal B-cell proliferation include ageing, posttransplant status, and HIV infection; however, recent reports [1, 13] have shown that EBV+ B cell neoplasms often occur in young patients without any known immunodeficiency. The etiology of EBV+ B-cell proliferation in these patients remains unknown, although a tolerogenic immune state has been reported as a possible cause [13].

IgG4-RD is usually treated by administration of corticosteroids [14], but a standard treatment has not been established for patients who are resistant to corticosteroids or require long-term administration of high doses of corticosteroids. It has also been reported that rituximab is effective for some patients with IgG4-related disease, including steroid-resistant cases [15, 16]. Rituximab probably depletes B cells as a precursor to IgG4-producing plasmacytoid cells [16, 17]. In addition, the fact that 58 % of cases with IgG4-related lymphadenopathy harbor EBV-infected B cells [6] gives rise to the possibility that IgG4-RD is often unexpectedly affected by EBV infection. These EBV-infected cells can be targeted by rituximab if they possess CD20. In accordance with this information, we administered rituximab-based immunotherapy with the expectation of targeting both IgG4-RD and B-cell LPD. However, it remains unclear whether these EBV-infected B cells acquire clonality and dominancy that arises in IgG4-producing plasmacytoid cells, or B-cell neoplasms such as DLBCL. In the present case, the clonality of EBV-infected B cells was not clear, but plasma EBV DNA was detected at high levels with high titers of EBV VCA and EA antibodies, indicating chronic EBV infection. It is well known that such EBV-infected B cells can develop B-cell LPDs with both CD20 and CD30 expression in immunodeficient conditions such as aged, posttransplant, or HIV virus-infected status [18–20]. Thus, the fact that large lymphoid CD20^+^ cells in the present case co-expressed CD30 may suggest an immunodeficient condition in which the etiologic role of IgG4-producing plasmacytoid cells is unknown. Of note, older patients with EBV-associated DLBCL and CD30 expression have a very poor outcome [9]; however, rituximab-combined immunochemotherapy successfully eradicated the EBV-infected cells in our patient, as shown by the EBV DNA level.

EBV-infected germinal center B cells are considered to be the origin of EBV-related B-cell LPD [3]. In our patient, EBER^+^ cells proliferated in the interfollicular zone, similar to cases with EBV-related B-cell LPD. Thus, we decided to treat this patient with rituximab, because the eradication of EBV is reported to be important for a favorable prognosis in EBV-related B-cell LPD [21]. It seems that rituximab is effective for both CD20^+^ large lymphoid cells and IgG4-producing plasmacytoid cells in our patient, because rituximab-combined treatment resulted in complete disappearance of nodal and extranodal tumors with reduction of the serum plasma EBV DNA level, as serum IgG4 remains to be detected at a drastically decreased level. Notably, although our patient was administered corticosteroid transiently, the therapeutic effect has persisted as of 1 year after stopping corticosteroid treatment. We believe that rituximab was highly effective in part by eradicating the EBV-infected B cells, because the reduction of lymphadenopathy was much more extreme than extranodal lesions in most reported cases, which were administered rituximab for IgG4-related disease without evaluation for EBV infection [16]. However, in our case, extranodal lesions without evidence of EBV infection also responded to the rituximab therapy. The mechanism of the response is unclear, although changes in systemic immune function caused by eradication of EBV might have contributed to the reconstitution of extranodal lesions. The role of rituximab in the treatment of IgG4-RD with EBV-infected B cells should be further investigated in both clinical trials and basic studies.

It has been reported that an elevated sIL2R value (>1500 U/mL) indicates poor prognosis in a variety of B-cell lymphomas [22]. In our case, the sIL2R value was 2130 U/mL, which dropped to 395 U/mL after the treatment, possibly corresponding to the clinical effect of treatment with rituximab. However, the role of sIL2R elevation in EBV+ B-cell LPD should be studied further in the future, although the induction of T cells by tumor-associated macrophages has been implicated as a cause of such sIL2R elevation in B-cell lymphomas [22].

## Conclusions

Our case may contribute to understanding the association between IgG4-RD and EBV infection, and the development of a novel therapeutic strategy for IgG4-RD.

## Abbreviations

DLBCL, diffuse large B-cell lymphoma; EA, early antigen; EBER, EBV-encoded RNA; EBNA, EB nuclear antigen; EBV, Epstein-Barr virus; FDG, fluorodeoxy glucose; IgG4-RD, IgG4-related disease; LMP, latent membrane protein; LPD, lymphoproliferative disorders; PET-CT, positron emission tomography-computed tomography; sIL2-R, soluble-IL2 receptor; VCA, viral capsid antigen
